# Research on Diet and Human Health

**DOI:** 10.3390/ijerph19116526

**Published:** 2022-05-27

**Authors:** José Francisco López-Gil, Pedro Juan Tárraga-López

**Affiliations:** 1 Health and Social Research Center, Universidad de Castilla-La Mancha (UCLM), 16002 Cuenca, Spain; 2 Departamento de Ciencias Médicas, Facultad de Medicina, Universidad Castilla-La Mancha (UCLM), 02008 Albacete, Spain

One of the major public health challenges is the global burden and threat of noncommunicable diseases (NCDs) [1]. It is estimated that NCDs cause a total of 41 million deaths annually, which is equivalent to 71% of all deaths worldwide, including mainly cardiovascular diseases (17.9 millions of deaths), cancers (9.3 million), chronic respiratory diseases (4.1 million) and diabetes (1.5 million) [2]. These major NCDs encompass some behavioral risk factors, such as tobacco smoking, insufficient physical activity, alcohol consumption and, especially, inadequate dietary pattern [1].

Dietary pattern means the totality of what individuals usually eat and drink, and the parts of the pattern act synergistically to affect health [3]. As a consequence, the dietary pattern may offer a higher prediction of overall health status and disease risk than single nutrients or foods [3]. In this sense, the World Health Organization (WHO) advises to follow a healthy diet to combat malnutrition in all its manifestations, as well as NCDs, such as heart disease, cancer, stroke, and diabetes [4]. Strikingly, the need to improve dietary patterns at the worldwide level has recently been pointed out [5], since an inadequate dietary pattern is a risk factor that causes more deaths than other factors habitually considered, such as tobacco smoking [6]. Supporting this notion, offering support based on scientific evidence for healthier dietary patterns could play a crucial role in public health [1].

Following the WHO, a healthy diet for adults must include the following components [4]: (a) vegetables, fruit, nuts, legumes, and whole grains; (b) at least 400 g (i.e., five servings) of vegetables and fruit daily; (c) less than 10% of total calorie intake from free sugars; (d) less than 30% of total calorie intake from fats, of which less than 10% of total calorie intake from saturated fats and less than 1% of total calorie intake from trans-fats; and (e) less than 5 g of salt (equivalent to approximately one teaspoon) daily. Notwithstanding, the meaning of what involves a healthy diet is constantly changing to update the understanding of the evolving role of different essential nutrients, foods, and other factors in relation to human health and disease [7].

In relation to specific dietary patterns, there is continuing controversy regarding the comparative efficacy of popular/well-known dietary patterns in decreasing risk factors for the development NCDs [8,9,10,11,12]. Aggarwal et al. [12] recently highlighted that there is sufficient evidence to promote the adherence to whole food plant-based diets, Dietary Approaches to Stop Hypertension (DASH) dietary patterns, or the Mediterranean diet, while warning people of the danger of Paleolithic diets and very low carbohydrate/ketogenic diets. Trying to clarify this issue, Get et al. [13] performed a network meta-analysis comparing the dietary macronutrient patterns of 14 popular/well-known dietary patterns for cardiovascular risk factors and weight reduction in adults. These same authors found moderate evidence for most of the diets over six months, leading to significant improvements in cardiovascular risk factors (especially blood pressure) and modest evidence for weight reduction. However, at 12 months, the benefits on cardiovascular risk factors and the changes on weight loss substantially disappear.

Despite the above, there is a need for diet improvement at the worldwide, national, and regional level [5]. The Sustainable Development Goal (SDG) target 3.4 is to decrease premature deaths from NCDs by one-third by 2030 compared to 2015 rates, among other actions [14]. However, no country will be able to achieve SDG target 3.4 by addressing only one NCD [15]. Scientific evidence from clinical trials and epidemiological studies concludes that healthy dietary patterns decrease the risk of suffering a NCDs (e.g., cancer, cardiovascular disease) [8]. Supporting this notion, the evidence displays that dietary interventions are relevant cost-effective strategies since, if provided promptly to patients, they can diminish the requirement for higher cost treatments [2]. Therefore, effective intervention programs aimed to reduce NCD can be implemented through a primary health care approach to reinforce early screening and appropriate management [2]. Due to the fact that the improvement of dietary patterns may potentially avoid one out of every five deaths worldwide [5], it seems reasonable to highlight the urgent need to improve the human diet in all countries, as well as the need for further studies to address this global concern.

Based on the above, the aim of this Special Issue titled “Research on Diet and Human Health” is to provide a comprehensive overview of the role of diet in several human health outcomes, to update knowledge on the factors implied this relationship, and to report dietary actions that could be established to overcome human health diseases. Since the relationship between diet and human health is multidisciplinary, this Special Issue will also attempt to include various perspectives on how to approach this relationship in the clinical setting, being of interest to endocrinologists, primary care physicians, nutritionists and nurses, among others.

## Data Availability

Not applicable.
